# Acute Exposure of Apigenin Induces Hepatotoxicity in Swiss Mice

**DOI:** 10.1371/journal.pone.0031964

**Published:** 2012-02-16

**Authors:** Prabhat Singh, Shrawan Kumar Mishra, Sanjeev Noel, Sharad Sharma, Srikanta Kumar Rath

**Affiliations:** Division of Toxicology, Central Drug Research Institute (CSIR), Lucknow, India; Wayne State University School of Medicine, United States of America

## Abstract

Apigenin, a dietary flavonoid, is reported to have several therapeutic effects in different diseases including cancer. Toxicity of Apigenin is however, least explored, and reports are scanty in literature. This warrants dose-specific evaluation of toxicity *in vivo*. In the present study, Apigenin was administered intraperitoneally to Swiss mice at doses of 25, 50, 100 and 200 mg/kg. Serum levels of alanine amino transferase (ALT), aspartate amino transferase (AST) and alkaline phosphatase (ALP) were measured along with the examination of liver histology, reactive oxygen species (ROS) in blood, lipid peroxidation (LPO), glutathione level, superoxide dismutase activity, catalase activity, glutathione S-transferase activity and gene expression in liver tissue. Increase in ALT, AST, ALP, ROS, ratio of oxidized to reduced glutathione (GSSG/GSH) and LPO, altered enzyme activities along with damaged histoarchitecture in the liver of 100 or 200 mg/kg Apigenin treated animals were found. Microarray analysis revealed the differential expression of genes that correspond to different biologically relevant pathways including oxidative stress and apoptosis. In conclusion, these results suggested the oxidative stress induced liver damage which may be due to the regulation of multiple genes by Apigenin at higher doses in Swiss mice.

## Introduction

Apigenin (4′, 5, 7-trihydroxyflavone) has been shown to possess diverse therapeutic potentials [1]. It improves cancer cell response to chemotherapy [2] and prevents tumourogenic activities by inhibiting protein kinase, MAP Kinase or chronically activates PI3K-Akt mediated nuclear factor-kappa-B [3]. Apigenin modulates immune cells functioning, maintains immune cells in inflammation, autoimmunity or lymphoproliferation [3] and inhibits auto antigen-presenting cells necessary for activation and expansion of auto reactive Th1 and Th17 cells and B cells in lupus [4]. Vasorelaxing and anti-platelet activities of Apigenin have also been demonstrated [5]. Recently, Apigenin has drawn attention of Scientists for its use in therapeutics [6]. However, few reports demonstrated that Apigenin produces phenoxyl radicals [7] or reactive oxygen species [8], [9] and induces cytotoxicity [10] or clastogenicity [11] in different *in vitro* models. Chemical Selection Working Group of FDA, USA (2000) recommends developmental toxicity and chromosomal aberration assays for Apigenin. This study explored its toxicity on mice liver following single intraperitoneal exposure.

## Materials and Methods

### Animals and drug administration

10–12 weeks old male Swiss mice, weighing 25–30 g were obtained from Laboratory Animal Division of CDRI following Institutional Animals Ethics Committee clearance (114/07/Toxicol./IAEC) and randomly allocated to the following groups containing eight animals each.

Group I: Vehicle (100 µl DMSO) controls
Group II: 25 mg/kg Apigenin
Group III: 50 mg/kg Apigenin
Group IV: 100 mg/kg Apigenin
Group V: 200 mg/kg Apigenin

Animals were treated with Apigenin once and sacrificed 24 hrs after the treatment. Animals were maintained in optimal conditions of temperature (25±2°C) and 12 hrs light/dark cycles and fed with standard pelleted diet and water *ad libitum*. Animal ethics guidelines were followed in all animal procedures. Apigenin was administered intraperitoneally as was done in the previous studies [4], [6].

### Blood collection, serum biochemistry and ROS estimation

At autopsy blood was withdrawn from each animal by cardiac puncture and allowed to stand undisturbed for 30 min. Serum was separated and levels of ALT, AST and ALP were estimated using an automated biochemical analyzer (Beckman Coulter, USA). Intracellular ROS in peripheral blood mononuclear cells (PBMC) were analyzed using fluorescent probe 2′, 7′-dichlorofluorescein-diacetate, a non-fluorescent compound under normal condition, which is converted into highly fluorescent dichlorofluorescein (DCF) by cellular peroxides. Cell-associated fluorescence was monitored on fluorescence activated cell sorter (Beckman Coulter, USA).

### Liver tissue biochemistry

Liver tissue homogenate was used for antioxidant enzymatic assays. Malondialdehyde (MDA) concentration (a measure of Lipid peroxidation; LPO) and antioxidant enzymes activities (superoxide dismutase, catalase, glutathione peroxidase, glutathione S-transferase) were estimated using standard tests [12]–[16]. GSH and GSSG contents were estimated following the instruction of Glutathione Assay Kit (BioVision, CA, USA). Total protein content was estimated according to Lowry et al. [17] using Bovine Serum Albumin as a standard.

### Liver histology

Liver tissue was fixed in 10% buffered formalin for histological investigations. Fixed liver tissues were washed overnight, dehydrated through graded alcohols and embedded in paraffin wax. Serial sections of about 5 µm thickness were stained with hematoxylin and eosin (H&E) for histological examinations.

### RNA isolation, Microarray, Clustering and GenMAPP analysis

Total RNA was isolated from 50 mg of frozen liver and quantified by spectrophotometer and formaldehyde gel electrophoresis. RNA samples with approximately 2∶1 ratio of 28 S∶18 S rRNA and 260/280 values≥1.8 were used for gene expression analysis. The methodology of microarray experiments was according to Noel et al. [18]. 22.4 k mouse arrays (http://www.microarrays.ca) containing 23041 unique probes were used. Raw intensity data was analyzed with Avadis Express version 4.3 (Strand life Sciences, India) and the background corrected intensities were LOWESS normalized (Cy5 against Cy3) to obtain log (base 2) ratios. Statistically significant difference between controls and Apigenin treated mice was deduced with two sample t-test and probes with p<0.05 and 2-fold differential expression at 25, 50, 100 mg/kg doses were identified. Raw and log transformed data (series accession no. GSE 12716) has been submitted to Gene Expression Omnibus database (www.ncbi.nlm.nih.gov/geo/) and conforms to MIAME guidelines developed by microarray gene expression data (MGED) society. Clustering techniques have been applied for the identification of patterns in gene-expression. Intensity values of duplicate spots were averaged in order to get a single mean value to perform k-means clustering with MeV version 3.1 [TM4, The Institute of Genomic Research]. Each expression cluster was further clustered hierarchically with Euclidean distance matrix and average linkage to identify gene with similar expression patterns. Gene expression data of 25, 50, 100 mg/kg doses were separately listed to make a representative gene-expression dataset for identifying affected pathways using GenMAPP version 2.1. Moreover, GenMAPP gene expression dataset file (.gex) was exported to MAPPFinder to calculate the percentage of genes with significant expression change, statistical score for each Gene Ontology (GO) term and Z score.

### Quantitative real time PCR analysis

Real-time PCR was performed according to the supplier protocol (Invitrogen, California, USA). Reactions were run in Light Cycler 480 system using forward and reverse primers (Table 1 and Table S1) and analyzed by LightCycler® 480 Software release 1.5.0. Samples were pooled group wise and experiments were carried out in triplicates. *β-actin* was used as an internal control. Melting curve analysis was performed for each primer pair and relative change in mRNA level between control and treated groups were calculated by using 2^−ΔΔCT^ method.

**Table 1:** List of primers used in Quantitative Real-time polymerase chain reaction.

Gene	Forward primer (5′-3′)	Reverse primer (5′-3′)
*Actb*	GGCTGTATTCCCCTCCATCG	CCAGTTGGTAACAATGCCATGT
*Sod1*	TTTTTGCGCGGTCCTTTCCTG	GGTTCACCGCTTGCCTTCTGCT
*Cat*	AGCGACCAGATGAAGCAGTG	TCCGCTCTCTGTCAAAGTGTG
*Gpx1*	ATGTCGCGTCTCTCTGAGG	CCGAACTGATTGCACGGGAA
*Gsta4*	GCTGCGGCTGGAGTGGAGTTT	GGATGGCCCTGGTCTGTGTCA
*Hsc70*	CCGATGAAGCTGTTGCCTATGGT	GTGTCTGCTTGGTGGGGATGGT
*Neo1*	CATTGTGGTCCGAGGTTATGC	GGCACTGGAGTGATGGAGC
*Zfp110*	GCACTGGAAAGAGGAAAGGCA	CTGCTCAGAACCCTGTTGCT
*Erp29*	GGGCAGTTAAGGTTGGAGCC	AGGATCTTCCCCATGATCTTCA
*Idh3a*	TGGGTGTCCAAGGTCTCTC	CTCCCACTGAATAGGTGCTTTG
*Duox1*	CCTGGTTGGGACACTGGCTTCTT	GTGTCGGGGGTTAGGCAGGTAGG
*Clca1*	AGCCCTCATAGAAGCTGAACA	CGCACTTTTAGGCTGTATCTACC

Note: List of other primers used in Quantitative Real-time PCR is given in Table S1.

### Western blot analysis

Proteins were isolated from liver tissue using the modified protocol of Ghribi et al., 2001 [19]. Tissues from control and various treatment groups were homogenized with 5–10 volumes of lysis buffer (200 mM HEPES, 10 mM KCl, 1.5 mM MgCl_2_, 1 mM EDTA, 1 mM EGTA, 1 mM DTT, 2 mM PMSF, 1× Protease inhibitor cocktail). Cellular debris were spun down at 20,000×g for 30 min in 4°C and supernatants were used as whole protein extract. Isolated proteins were quantified using Bradford reagent. 50 µg protein from each sample was separated on 15% SDS-PAGE and transferred on to a nitrocellulose membrane using a semi-dry electro blotting apparatus (GE Healthcare, UK). Transfer was examined by Ponceau S stain and washed with triple distilled water until the stain disappeared. Membrane was blocked overnight in 5% Non-Fat dried milk at 4°C. Membrane was washed with 0.1% PBST and probed with primary antibodies (Actin, SOD1 and Hsp70). After primary antibody incubation further washing was done in 0.1% PBST. Membrane was incubated in HRP conjugated secondary antibody and washed again. Enhanced chemi-luminescent detection system (GE Healthcare, UK) was used to develop the blots. Blots were further used for densitometric analysis and normalization.

### Statistical analysis

Data were expressed as mean±standard error of the means wherever required. Group means were compared by one-way analysis of variance (ANOVA) followed by Newman-Keuls Multiple Comparison Test. p<0.05 was considered significant.

## Results

### Serum ALT, AST and ALP

**Figure 1:**
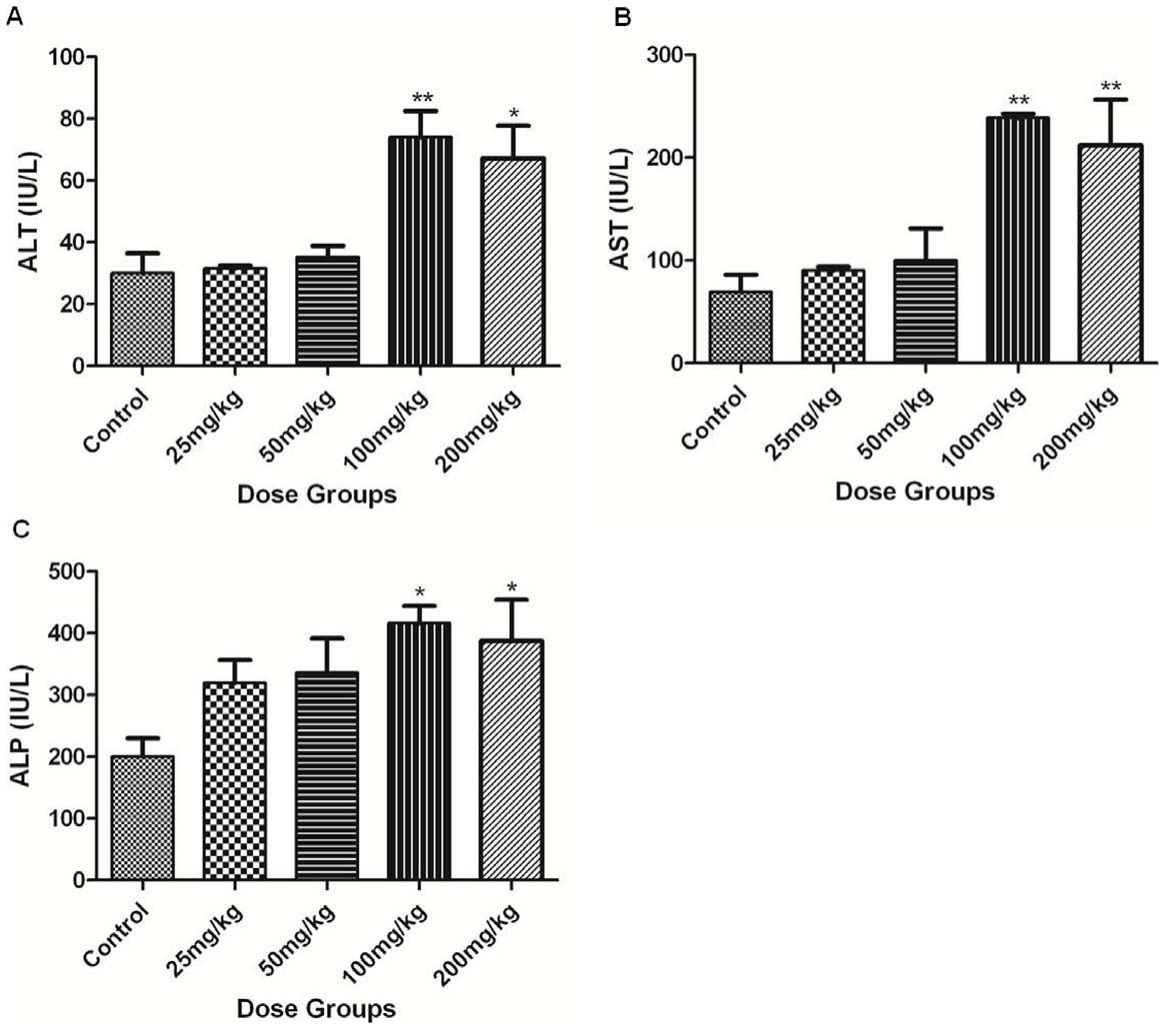
Levels of serum biomarkers of hepatotoxicity. (A) Alanine amino transferase (ALT), (B) Aspartate amino transferase (AST), and (C) Alkaline phosphatase (ALP) levels were estimated in serum following the administration of Apigenin at different doses (Control, 25, 50, 100 and 200 mg/kg). The asterisks indicate significance of differences (*-p<0.05; **-p<0.01; ***-p<0.001) in comparison to control.

Serum ALT, AST and ALP were unaltered in 25 or 50 mg/kg dose groups as compared to control. A significant increase in serum ALT (100 mg/kg; p<0.01 and 200 mg/kg; p<0.05), AST (100 and 200 mg/kg; p<0.01) and ALP (100 and 200 mg/kg; p<0.05) was observed in animals belonging to the higher dose groups (Figures 1 A–C).

### ROS generation

**Figure 2:**
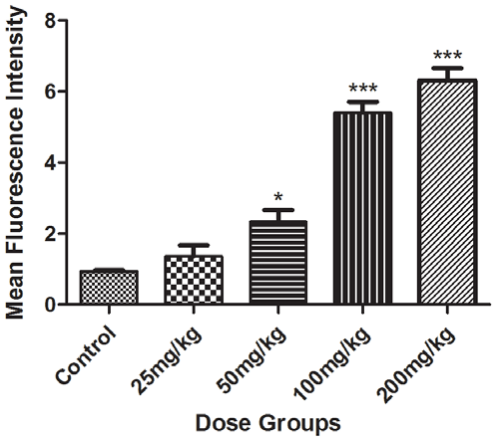
Mean fluorescence intensity of dichlorofluorescein (DCF). Intracellular ROS in peripheral blood mononuclear cells (PBMC) were analyzed in Apigenin treated animals at different doses (Control, 25, 50, 100 and 200 mg/kg) using fluorescent probe 2′, 7′-dichlorofluorescein-diacetate. The asterisks indicate significance of differences (*-p<0.05; ** -p<0.01; ***-p<0.001) in comparison to control.

No significant DCF peak shift was observed in 25 mg/kg Apigenin treated group. The oxidized dichlorofluorescein (DCF) peak shifts were 2.24 (p<0.05), 5.76 and 6.56 (p<0.001) fold in 50, 100 and 200 mg/kg dose groups respectively as compared to controls (Figure 2 and Figure S1).

### MDA concentration

**Figure 3:**
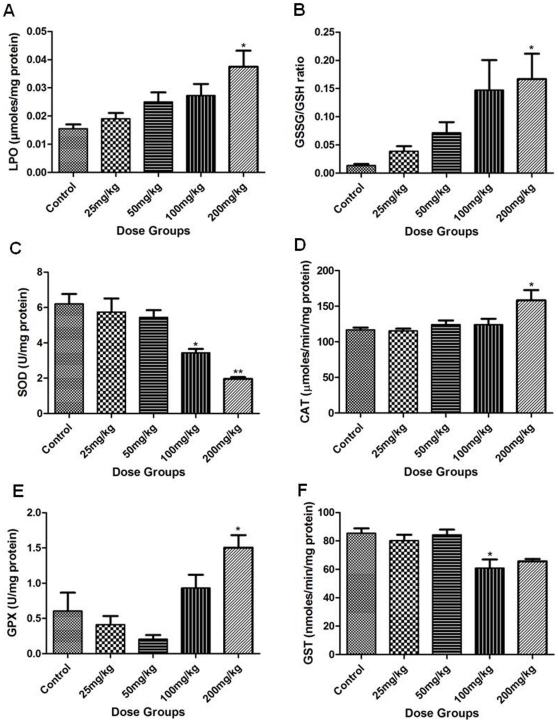
Level of oxidative stress parameters. (A) Lipid peroxidation level, (B) Ratio of GSSG/GSH, (C) activity of SOD, (D) activity of CAT, (E) activity of GPx, and (F) activity of GST were estimated in liver following the administration of Apigenin at different doses (Control, 25, 50, 100 and 200 mg/kg). The asterisks indicate significance of differences (*-p<0.05; **-p<0.01; ***- p<0.001) in comparison to control.

MDA concentration in the liver of 25 and 50 mg/kg groups was unaltered. Significant increase in MDA concentration was found in 200 mg/kg (p<0.05) group (Figure 3-A). An increase in MDA concentration was found in 100 mg/kg Apigenin treated animals, which was statistically non-significant as compared to controls.

### GSSG and GSH ratio

A trend of increase in GSSG/GSH ratio was found along the group (25 mg/kg to 200 mg/kg) of Apigenin and it was significantly increased in 200 mg/kg group (p<0.05; Figure 3-B). GSH in liver tissue of 200 mg/kg group was significantly decreased (p<0.05) but not in other groups (25, 50 or 100 mg/kg) (Figure S2). A trend of dose dependent increase in GSSG was observed along the groups (Figure S3).

### SOD activity and expression

**Figure 4:**
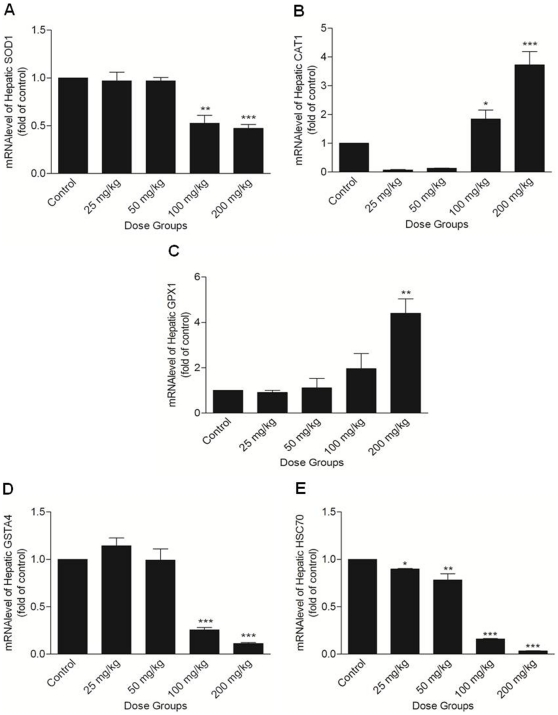
Quantitative real time PCR analysis of stress regulated genes. (A) SOD1, (B) CAT, (C) GPx1, (D) GSTA4, and (E) Hsc70 mRNA levels in the liver of mice treated with different doses of Apigenin (Control, 25, 50, 100 and 200 mg/kg). The asterisks indicate significance of differences (*-p<0.05; **-p<0.01; ***-p<0.001) in comparison to control.

**Figure 5:**
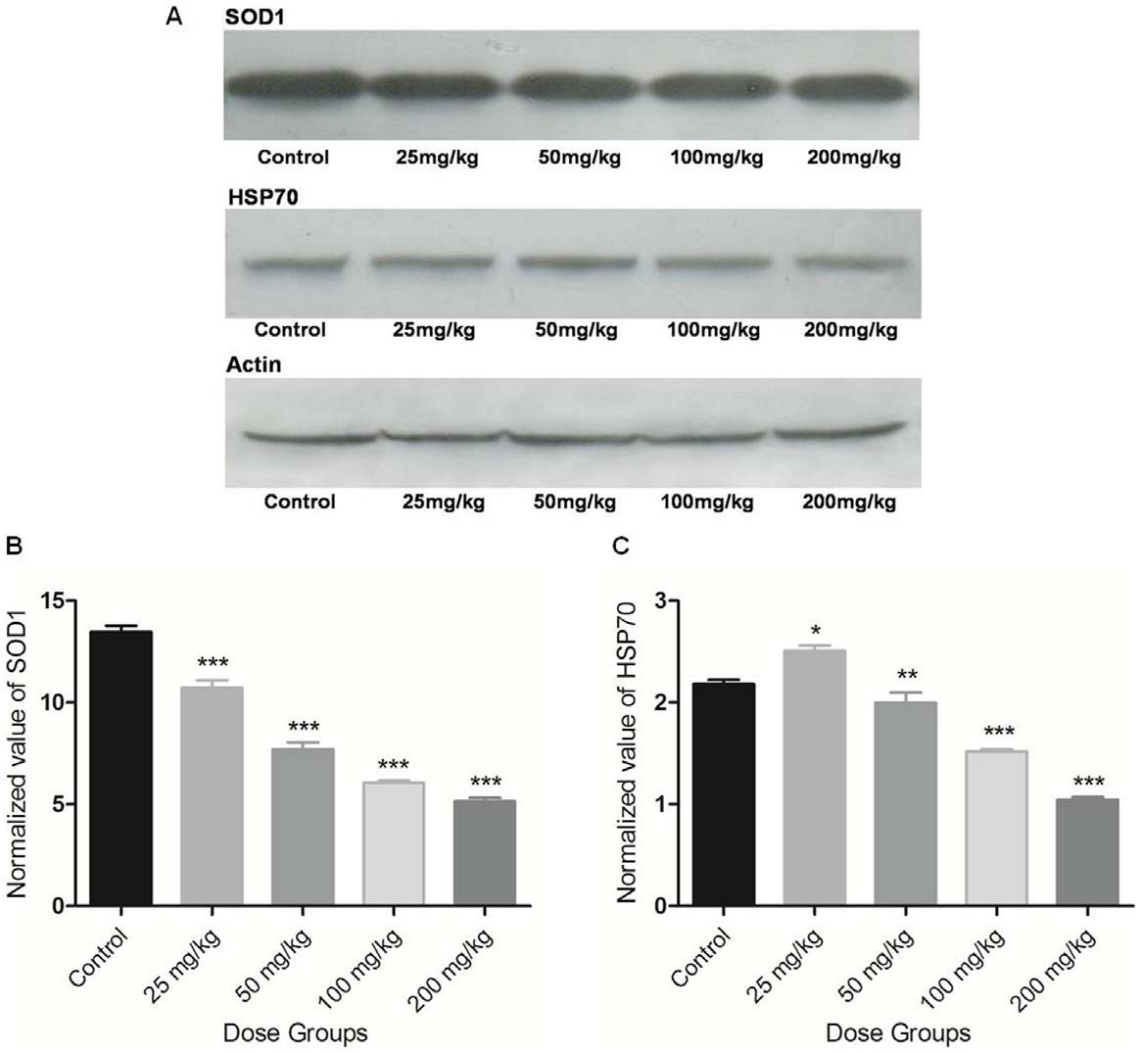
Western blot analysis of SOD1 and Hsp70. (A) Western blots of SOD1, Hsp70 and Actin genes. Relative band intensity value of (B) SOD1, and (C) Hsp70 after normalization with Actin. The asterisks indicate significance of differences (*-p<0.05; **-p<0.01; ***-p<0.001) in comparison to control.

SOD activity and mRNA level were unaltered in the lower treatment groups of Apigenin (25 and 50 mg/kg) as compared to controls. Higher doses of Apigenin significantly reduced the activity (100 mg/kg; p<0.05 and 200 mg/kg; p<0.01; Figure 3-C) and expression of SOD measured at transcript (Figure 4-A) and protein (Figures 5-A and B) level as compared to controls.

### CAT, GPx, GST activities and mRNA level

CAT, GPx activities and mRNA level did not change in lower treatment groups (25 and 50 mg/kg) of Apigenin as compared to control. In 100 mg/kg Apigenin treated group, increase in CAT (p<0.05) and GPx (not significant) mRNA level was observed. CAT, GPx activities (Figures 3-D and E) and their mRNA levels (Figures 4-B and C) were significantly increased in highest dose group (200 mg/kg; p<0.05). In lower treatment groups of Apigenin (25 and 50 mg/kg), activity and mRNA level of GST did not change as compared to control. Activity of GST (Figure 3-F) was decreased in both the higher treatment groups (100 and 200 mg/kg, statistically significant only in 100 mg/kg Apigenin treatment group at p<0.05). Reduction in its mRNA level in higher treatment groups (100 and 200 mg/kg) was statistically significant (p<0.001) (Figure 4-D).

### Hsp70 expression

Hsp70 mRNA level showed a significant decrease along the Apigenin treated groups (25 mg/kg; p<0.05 or 50 mg/kg; p<0.01), decrease was apparent in higher treatment groups (100 and 200 mg/kg; p<0.001) as compared to control (Figure 4-E). mRNA level of other members of Hsp70 family were also decreased in higher treatment groups of Apigenin (100 and 200 mg/kg; Figure S4). Hsp70 protein content was decreased significantly in 100 and 200 mg/kg Apigenin treated groups (p<0.001) (Figures 5-A and C). A significant change in Hsp70 protein content was observed in lower treatment groups (25 mg/kg; p<0.05 and 50 mg/kg; p<0.01) of Apigenin as compared to control.

### Liver histology

**Figure 6:**
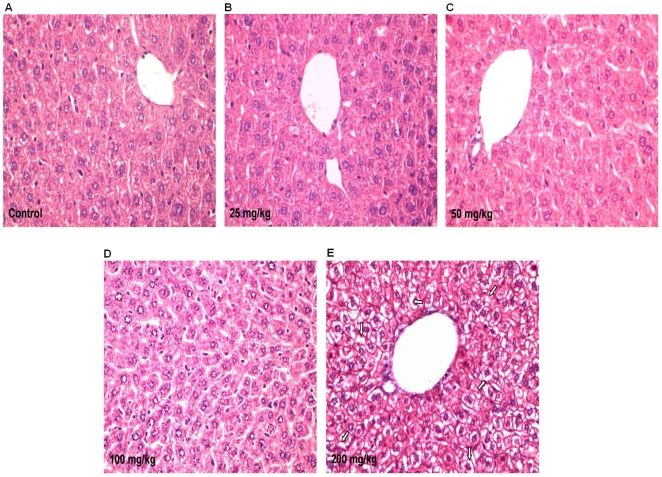
Histological examination of liver sections. Photomicrographs of transverse section of mice liver (A) Control, (B) 25 mg/kg, (C) 50 mg/kg Apigenin treated groups. Slight hydropic changes and degeneration of cytoplasm within hepatocytes were observed in (D) 100 mg/kg, and (E) 200 mg/kg Apigenin treated groups respectively. Arrows indicated the degenerated hepatocytes in 200 mg/kg Apigenin treated group.

Well distributed normal hepatocytes with central vein, bile duct and hepatic artery were observed in 25 and 50 mg/kg groups. In 100 mg/kg dose group hydropic changes were observed, these changes were eminent with ballooning and degeneration of hepatocytes in 200 mg/kg dosed animals (Figures 6 A–E).

### Differential gene expression and pathway identification

**Figure 7:**
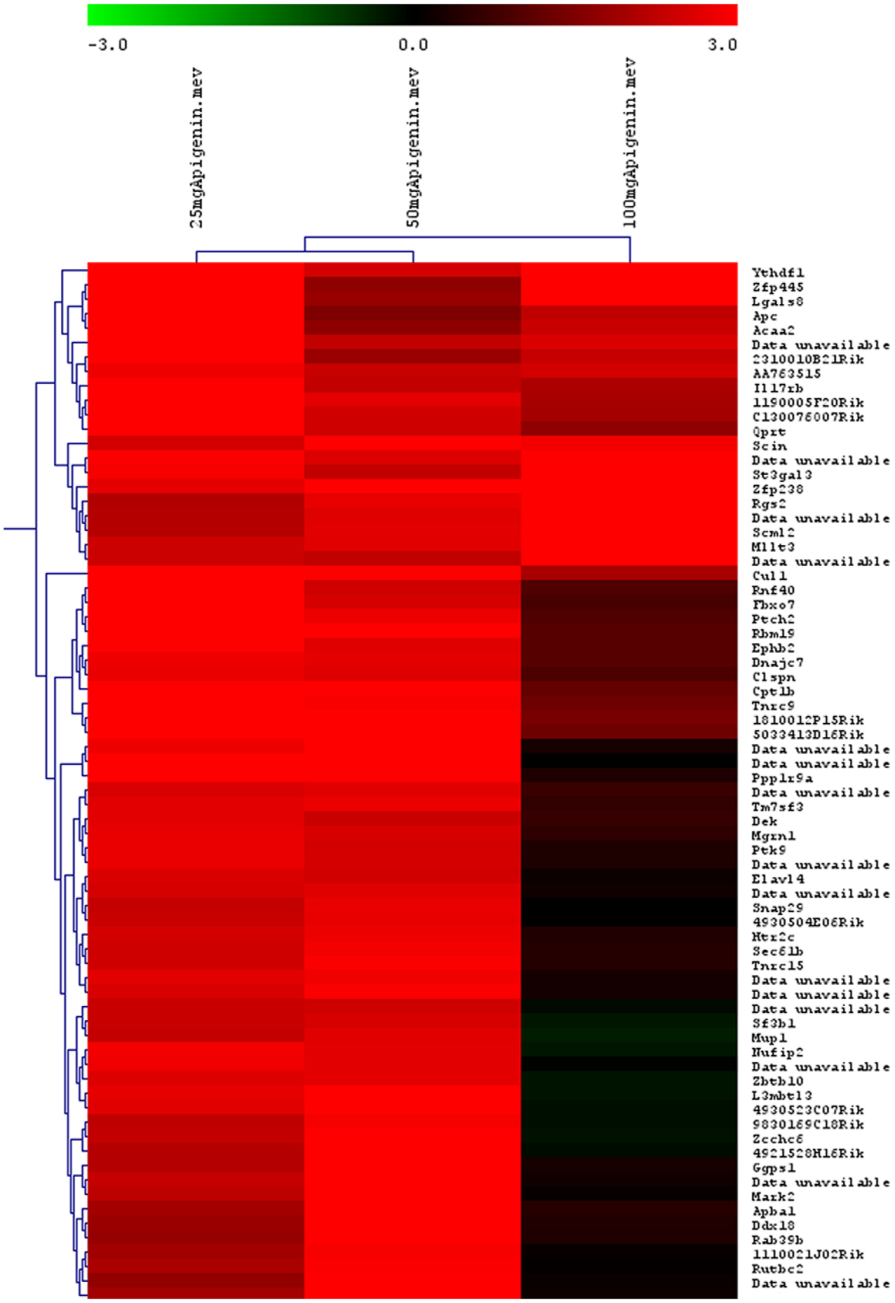
Cluster analysis of differentially expressed genes. K-mean and hierarchical clustering identified the similar pattern of expression in different dose groups of Apigenin (25, 50, 100 mg/kg). In heat map, intensities of red and green colours were proportional to relative gene induction and repression, respectively.

48 differentially expressed genes (36 up-regulated and 12 down-regulated; Table 2) were identified. Among them few genes have not been assigned any biological function, so far. Real time PCR analysis of selected genes (Table 1 and Table S1) showed the similar trend as found in microarray results. K-mean and hierarchical clustering identified the similar pattern of expression in genes at different dose levels (Figure 7). Major pathways that showed Apigenin induced perturbations include oxidative stress, apoptosis, inflammatory and MAP Kinase related pathways. Microarray data analysis with MAPPFinder revealed the genes involved in enzyme activities, cell proliferation, metabolic processes, cell structure and signal transduction related pathways were most affected with increased significant Z score (Z score>2) (Table 3 and Table S3).

**Table 2:** List of differentially expressed genes identified at p<0.05 and two fold change.

Gene	Unigene ID	Function	Regulation	P value	Log value	Std dev#
*Neo1*	Mm.42249	ATP binding, cadherin binding	Up	0.015	1.185	0.995
*Bnip3l*	Mm.29820	Positive regulator of apoptosis, integral to membrane	Up	0.027	1.180	1.123
*Prpsap1*	Mm.25125	Extracellular space, nucleotide biosynthesis	Up	0.013	1.099	0.901
*Eif5b*	Mm.260943	GTP binding, transferase activity, translation initiation factor activity	Up	0.006	1.247	0.881
*Polr2h*	Mm.288730	DNA-directed RNA polymerase I complex, core complex	Up	0.013	1.096	0.899
*Zfp110*	Mm.292297	DNA binding, receptor activity	Up	0.034	1.373	1.374
*Idh3a*	Mm.279195	Oxidoreductase activity	Down	0.016	−1.052	0.892
*Ltbp1*	Mm.269747	Calcium ion binding	Down	0.017	−1.566	1.349
*Clca1*	Mm.275745	Integral to plasma membrane	Down	0.035	−1.053	1.060
*Pank2*	Mm.101264	Pantothenate kinase activity	Down	0.042	−1.067	1.122
*Duox1*	Mm.108582	Calcium ion binding, electron carrier activity	Down	0.001	−1.084	0.579
*Pkmyt1*	Mm.182193	Protein amino acid phosphorylation	Down	0.013	−1.035	0.844

# Standard deviation.

Note: Complete list of differentially expressed genes at p<0.05 and two fold change is given in Table S2.

**Table 3:** List of most affected Gene Ontology (GO) terms (Z score>2).

Gene Ontology ID	Gene Ontology name	Z score	Permute P
**Probes involved in biological processes**			
22008	Neurogenesis	2.916	0.007
9987	Cellular process	2.601	0.012
6412	Translation	2.399	0.014
43170	Macromolecule metabolic process	2.263	0.014
7067	Mitosis	2.366	0.019
9888	Tissue development	2.366	0.020
904	Cellular morphogenesis during differentiation	2.410	0.026
45045	Secretory pathway	2.366	0.026
87	M phase of mitotic cell cycle	2.288	0.027
**Probes involved in molecular functions**			
4930	G-protein coupled receptor activity	2.410	0.015
30528	Transcription regulator activity	2.431	0.018
16887	ATPase activity	2.203	0.019
3713	Transcription coactivator activity	2.521	0.022
17111	Nucleoside-triphosphatase activity	2.372	0.022
30246	Carbohydrate binding	2.512	0.023
3712	Transcription cofactor activity	2.130	0.023
16817	Hydrolase activity\, acting on acid anhydrides	2.367	0.025
5529	Sugar binding	2.591	0.033
**Probes involved in cellular components**			
5788	Endoplasmic reticulum lumen	9.883	0.014
30529	Ribonucleoprotein complex	2.743	0.006
5829	Cytosol	2.680	0.012
5746	Mitochondrial respiratory chain	2.950	0.028

Note: Complete list of most affected Gene Ontology (GO) terms (Z score>2) is given in Table S3.

## Discussion

Apigenin at doses of 25, 50, 100 and 200 mg/kg were evaluated following acute exposure through intraperitoneal route to understand the dose dependent effects in Swiss mice. Intraperitoneal route of exposure enables the maximum bioavailability of Apigenin in liver. Doses of Apigenin were equivalent to the human exposure of flavones [20] based on the equivalent body surface area index [21]. Male Swiss mice were used in the present study to avoid any sex dependent variations in toxic effects in female mice due to the estrogenic action of Apigenin [22].

Unaltered serum ALT, AST and ALP in 25 or 50 mg/kg Apigenin doses indicate its non toxic effect at these doses. Significantly increased serum ALT, AST and ALP in 100 and 200 mg/kg Apigenin treated groups indicate the insults to liver as increased ALT, AST and ALP in serum are typical indicators of damaged liver [23]. Galati et al. [24] also reported 4-fold increased plasma ALT in CD-1 mice following 24 hrs of intraperitoneal injection of flavonoids like EGCG, propyl gallate, gallic acid and tannic acid. Normal liver histoarchitecture of 25 or 50 mg/kg Apigenin treated animals supports the serum findings and suggestive of non toxic effects at these doses. Hydropic changes along with ballooning and degeneration of hepatocytes in 100 and 200 mg/kg Apigenin treated groups are the signs of adverse effects on mouse liver. Five fold increased ROS level in PBMCs may be causative of damaged liver in 100 and 200 mg/kg Apigenin treated animals as ROS damages essential biological molecules like proteins, DNA and lipids. Previous studies also demonstrated ROS production by Apigenin [8], [9].

LPO is initiated by the attack of free radicals on fatty acid or fatty acyl side chain of any chemical entity and is regarded as one of the basic mechanism of tissue damage [25]. The increase of LPO level in Apigenin treated mice at 100 and 200 mg/kg indicates free radical generation showing the pro-oxidant nature of Apigenin. Similar nature of Apigenin is also demonstrated in the presence of high iron concentration in rat hepatocytes [26]. Decreased GSH and increased ratio of GSSG and GSH in mice liver further supports this view. Similar observations were made by Kachadourian and Day [27] in PC3 cells following Apigenin treatment. GSH is the functional anti-oxidative system in physiological conditions; its depletion might be due to its direct involvement in scavenging ROS in the process of neutralization and subsequent protection of essential thiol groups from oxidation. ROS are scavenged by cellular antioxidant defence system which includes intracellular enzymes such as SOD, CAT, GPx and GST. SOD activity and expression was decreased significantly following 100 and 200 mg/kg Apigenin doses. As SOD dismutates superoxide into oxygen and H_2_O_2_ provides an important antioxidant defence in cells exposed to oxygen, its decrease infers excessive ROS generation. Significantly increased CAT activity in 200 mg/kg Apigenin treated mice clearly indicates H_2_O_2_ generation. Unaltered CAT in mice at 100 mg/kg dose may be due to more turnover of CAT in cells following Apigenin exposure. CAT is solely responsible for the destruction of H_2_O_2_ while GPx has a wide spectrum of activity and reduces lipid peroxides. In the lower dose groups (25 and 50 mg/kg) CAT and GPx activities and mRNA level were not increased which might be due to insufficient ROS production in mice liver. GPx level was significantly increased in 200 mg/kg Apigenin treated mice which might be the result of decrease in GSH content. Decreased GST at 100 and 200 mg/kg Apigenin treated groups is in accordance with the findings of Sahu and Gray [28] who reported the flavonoid induced concentration-dependent decrease in GST activity. GST protects cells against toxicants by conjugating them to GSH, thereby neutralizing their electrophilic sites, and increases their solubility in aiding excretion from cells [16]. The decrease in GST along with SOD indicates severe insult to liver tissue following acute exposure of Apigenin at higher doses. Increase in GSSG and GSH ratio further indicated a shift of biological system towards the state of apoptosis or necrosis. Dose-dependent reduction in Hsp70 mRNA and protein was observed following Apigenin treatment. Hsp70 is a multigene family and it is expressed in different isoforms in which Hsc70/Hspa8 is constitutively expressed [29]. Hsp70 is involved in the regulation of cell proliferation, differentiation and can be induced by heat stress, hypoxia, metals or amino acid analogs exposure [29]. Previous studies revealed that Hsp synthesis is blocked following the treatment of other flavonoid such as Quercetin [30], [31]. Dose related decrease in Hsp70 expression indicates the involvement of heat shock and stress pathway that leads towards apoptosis [32].

Gene expression data provides insight into the ongoing molecular activities inside the cells especially in short term acute toxicity studies where the full phenotypic signs and symptoms may have not been fully developed [18]. In the present study, 48 differentially regulated genes were identified that are involved in important biological functions. Most of them (*Bnip3l*, *Neo1*, *Clca1*, *Idh3a*, *Pank2*, *Prpsap1*, *Eif5B*, *Polr2h*, *Zfp110*) are engaged in regulation of apoptosis, stress and cell growth. Isocitrate dehydrogenase (*Idh3a*) protects cell against oxidative damage [33] has been shown to be more active in producing Nicotinamide Adenine Dinucleotide Phosphate Reduced (NADPH) than other enzymes in the previous studies [34]. Down regulation of this gene clearly indicated that cell might have undergone oxidative stress following Apigenin treatment. Interestingly, the simultaneous upregulation of BCL2/adenovirus E1B interacting protein 3-like (*Bnip3l*) [35], and Neogenin (*Neo1*) [36] genes that are reported to regulate apoptosis, might be involved in the induction of apoptosis in degenerated hepatocytes of Apigenin treated mice in higher dose groups. Apigenin is reported to induce apoptosis by activating different genes like PKC-δ and caspases [2]. Apigenin upregulates the expression of genes involved in transcription and translation machinery; Phosphoribosyl pyrophosphate synthetase associated protein 1 (*Prpsap1*), Eukaryotic translation initiation factor 5B (*Eif5B*), DNA directed polymerase (*Polr2h*), Zinc finger protein 110 (*Zfp110*). Pantothenate kinase 2 (*Pank2*), a mitochondrial enzyme catalyses the first regulatory step of Coenzyme A synthesis, is found to be down regulated in present study. This gene is responsible for a genetic movement disorder named Pank-associated neurodegeneration. Recent evidences suggest the silencing of *Pank2* gene is directly associated with cell growth reduction and iron deregulation in hepatic cell lines [37]. Another downregulated gene was calcium-activated chloride channel (*Clca1*) which is integrated to plasma membrane. Another downregulated gene was calcium-activated chloride channel (*Clca1*) which is integrated to plasma membrane. Differential regulation of *Clca1* in normal, apoptotic and transformed mouse cells suggested its proapoptotic and antineoplastic nature [38]. Apigenin appears to affect the calcium ion homeostasis by modulating the expression of calcium ion binding proteins (Latent transforming growth factor beta binding protein; *Ltbp1* and Dual oxidase 1; *Duox1*). Further analysis of datasets on MAPPFinder identified GO terms (Z score>2) corresponding to various biological processes, molecular functions and cellular components. This provides evidences of significant change in gene expression following oxidative stress associated hepatotoxicity. Few identified genes have not been assigned any cellular functions that might be playing important role in Apigenin induced perturbations in mice liver.

Results indicate that Apigenin induces oxidative stress through different pathways ensuing liver toxicity. However, further studies are required to elucidate the detail molecular pathways of Apigenin action.

## Supporting Information

Figure S1 **Reactive oxygen species generation with maximum DCF peak shifts in 100 and 200 mg/kg Apigenin treated groups.**
(DOC)

Figure S2 **Decrease in reduced Glutathione content in 200 mg/kg Apigenin treatment group.**
(DOC)

Figure S3 **Increase in Oxidized Glutathione content (GSSG) along the Apigenin treatment groups.**
(DOC)

Figure S4 **Decrease in mRNA level of different members of Hsp70 family in higher treatment group of Apigenin (100 and 200 mg/kg).**
(DOC)

Table S1 **List of other primers used in Quantitative Real-time PCR.**
(XLS)

Table S2 **Complete list of differentially expressed genes.**
(XLS)

Table S3 **Complete list of most affected Gene Ontology (GO) terms (Z score>2).**
(XLS)
