# Supplementary material for: Acute Exposure of Apigenin Induces Hepatotoxicity in Swiss Mice
Source: PLoS One. 2012 Feb 16;7(2):e31964. doi: 10.1371/journal.pone.0031964 (PMC3281105; doi:10.1371/journal.pone.0031964)
Supplement: Figure S1 — Reactive oxygen species generation with maximum DCF peak shifts in 100 and 200 mg/kg Apigenin treated groups. (DOC) [file pone.0031964.s001.doc]

**Supplemental Figure 1**

Supplemental Figure 1 is showing the oxidized DCF (Di-chloro fluorescein) peak, indicative peroxide content. (A) Control, (B) 25 mg/kg Apigenin, (C) 50 mg/kg Apigenin, (D) 100 mg/kg Apigenin, and (E) 200mg/kg Apigenin. Note the significant peak shift at Apigenin 50, 100 and 200 mg/kg doses.
