# Supplementary material for: Acute Exposure of Apigenin Induces Hepatotoxicity in Swiss Mice
Source: PLoS One. 2012 Feb 16;7(2):e31964. doi: 10.1371/journal.pone.0031964 (PMC3281105; doi:10.1371/journal.pone.0031964)
Supplement: Figure S2 — Decrease in reduced Glutathione content in 200 mg/kg Apigenin treatment group. (DOC) [file pone.0031964.s002.doc]

**Supplemental Figure 2**

Supplemental Figure 2 is showing the level of Reduced Glutathione content (GSH) which was decreased significantly in 200 mg/kg (p< 0.05) as compared to controls. Animals of other dose groups (25, 50 and 100 mg/kg) showed no alteration in the level of Reduced Glutathione in liver tissue. The asterisks indicate significance of differences (*-p<0.05; **-p<0.01; ***-p<0.001) in comparison to control.
