# Supplementary material for: Acute Exposure of Apigenin Induces Hepatotoxicity in Swiss Mice
Source: PLoS One. 2012 Feb 16;7(2):e31964. doi: 10.1371/journal.pone.0031964 (PMC3281105; doi:10.1371/journal.pone.0031964)
Supplement: Figure S3 — Increase in Oxidized Glutathione content (GSSG) along the Apigenin treatment groups. (DOC) [file pone.0031964.s003.doc]

**Supplemental Figure 3**

Supplemental Figure 3 is showing the trend of increase in Oxidized Glutathione content (GSSG) along the Apigenin treatment groups. As a result, GSSG/GSH ratio was significantly elevated in 200 mg/kg Apigenin treated animals as compare to control. Depletion in Reduced Glutathione and restoration in Oxidized Glutathione is associated with the generation of oxidative stress.
