# Supplementary material for: Acute Exposure of Apigenin Induces Hepatotoxicity in Swiss Mice
Source: PLoS One. 2012 Feb 16;7(2):e31964. doi: 10.1371/journal.pone.0031964 (PMC3281105; doi:10.1371/journal.pone.0031964)
Supplement: Figure S4 — Decrease in mRNA level of different members of Hsp70 family in higher treatment group of Apigenin (100 and 200 mg/kg). (DOC) [file pone.0031964.s004.doc]

**Supplemental Figure 4**

A B

C

D E

Supplemental Figure 4 is showing the effect of different doses (Control, 25, 50, 100 and 200 mg/kg) of Apigenin on mRNA levels of different genes of HSP70 multigene family in mouse. (A) HSPA5, (B) HSPA2, (C) HSPA1L, (D) HSP70.1, (E) HSP70.3. The asterisks indicate significance of differences (*-p<0.05; **-p<0.01; ***-p<0.001) in comparison to control.
